# Modeling and Design Optimization of a Rotational Soft Robotic System Driven by Double Cone Dielectric Elastomer Actuators

**DOI:** 10.3389/frobt.2019.00150

**Published:** 2020-01-10

**Authors:** Sophie Nalbach, Rukmini Manoz Banda, Sipontina Croce, Gianluca Rizzello, David Naso, Stefan Seelecke

**Affiliations:** ^1^Center for Mechatronics and Automation Technologies (ZeMA) gGmbH, Saarbrücken, Germany; ^2^Department of Systems Engineering, Saarland University, Saarbrücken, Germany; ^3^Department of Material Science and Engineering, Saarland University, Saarbrücken, Germany; ^4^Department of Electrical and Information Engineering, Polytechnic University of Bari, Bari, Italy

**Keywords:** dielectric elastomer, soft robot, multi-DOF, double cone actuator, simulation

## Abstract

Dielectric elastomers (DEs) consist of highly compliant electrostatic transducers which can be operated as actuators, by converting an applied high voltage into motion, and as sensors, since capacitive changes can be related to displacement information. Due to large achievable deformation (on the order of 100%) and high flexibility, DEs appear as highly suitable for the design of soft robotic systems. An important requirement for robotic systems is the possibility of generating a multi degree-of-freedom (MDOF) actuation. By means of DE technology, a controllable motion along several directions can be made possible by combining different membrane actuators in protagonist-antagonist configurations, as well as by designing electrode patterns which allow independent activation of different sections of a single membrane. However, despite several concepts of DE soft robots have been presented in the recent literature, up to date there is still a lack of systematic studies targeted at optimizing the design of the system. To properly understand how different parameters influence the complex motion of DE soft robots, this paper presents an experimental study on how geometry scaling affects the performance of a specific MDOF actuator configuration. The system under investigation consists of two cone DE membranes rigidly connected along the outer diameter, and pre-compressed out-of-plane against each other via a rigid spacer. The electrodes of both membranes are partitioned in four sections that can be activated separately, thus allowing the desired MDOF actuation feature. Different prototypes are assembled and tested to study the influence of the inner radius as well as the length of the rigid spacer on the achievable motion range. For the first experimental study presented here, we focus our analysis on a single actuation variable, i.e., the rotation of the rigid spacer about a fixed axis. A physics-based model is then developed and validated based on the collected experimental measurements. A model-based investigation is subsequently performed, with the aim of studying the influence of the regarded parameters on the rotation angle. Finally, based on the results of the performed study, a model-based optimization of the prototype geometry is performed.

## Introduction

In recent years, the idea of human-robot cooperation, in which robots support human workers by undertaking exhausting or harmful subtasks of their work, is becoming more and more relevant. If robots operating with high force levels have to share the same working environment with humans, it is of fundamental importance that they do not cause harm to the users. A potential way of addressing this problem consists of designing soft robots in which conventional metal parts are replaced by highly compliant materials (Albu-Schaffer et al., 2008; Laschi et al., 2017). Ideally, these soft materials must be able to sustain the structure of the robot, as well as to provide actuation and sensing capabilities. Most of the current solutions to this problem are based on a combination of flexible structures, e.g., springs, pneumatic actuators, electric motors (Robinson et al., 1999; Pratt and Krupp, 2004; Pan et al., 2015). A viable alternative for the design of soft robots is represented by smart materials like shape memory alloys (Laschi et al., 2012; Cianchetti et al., 2015; Villoslada et al., 2015), shape memory polymers (Shen et al., 2016), or electro-active polymers (Shintake et al., 2015; Godaba et al., 2016). Among those materials, dielectric elastomers (DEs) represent a class of electro-active polymers which appear to be particularly suitable for soft robotics applications. This is due to a unique combination of features such as large deformations, high flexibility, lightweight, low power consumption, and self-sensing (Carpi et al., 2008).

**Figure 1:**
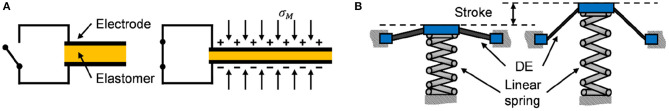
Structure of compliant capacitor and working principle of DE under high voltage activation **(A)**, and example of stroke generation via pre-stressed DEAs **(B)**.

A DE consists of a stretchable elastomer membrane that is sandwiched between two compliant electrodes. By applying high voltage to this soft capacitive structure, the charges distributing on the electrodes lead to a voltage-induced stress in the material typically referred to as Maxwell stress. The Maxwell stress, denoted by σ_*M*_, can be quantified as follows

(1) $$\begin{array}{l} \sigma_{M}=-\varepsilon_{0}\varepsilon_{r}E^{2}=\varepsilon_{0}\varepsilon_{r}{\left(\frac{v}{z}\right)}^{2}, \end{array}$$

where ε_0_ and ε_*r*_ denote the vacuum permittivity and the DE relative permittivity, respectively, *E* is the electric field in the DE material, *v* is the voltage applied between the electrodes, and *z* is the DE membrane thickness. As a result of the Maxwell stress, the DE membrane reacts with a reduction in thickness and, due to the incompressibility of the material, with an expansion of surface area (see Figure 1A). Both effects can be used to create the motion of an actuator.

To generate a stroke out of this surface change, a pre-load force must be applied to the DE membrane. This pre-stress can be realized in many different ways, e.g., via a linear spring (He et al., 2010), a bi-stable spring (Hodgins et al., 2013), a permanent magnet (Loew et al., 2018), or also another DE membrane (Cao et al., 2019). The working principle of a DE actuator (DEA) is shown in Figure 1B, in which the biasing element (i.e., a linear spring) creates a one-dimensional stroke when the membrane surface increases due to a voltage. The resulting stroke can be used in applications like pumps (Loverich et al., 2006; Carpi et al., 2010), valves (Goulbourne et al., 2004; Giousouf and Kovacs, 2013), or positioners (Jordan and McCarthy, 2011; Hau et al., 2012).

**Figure 2:**
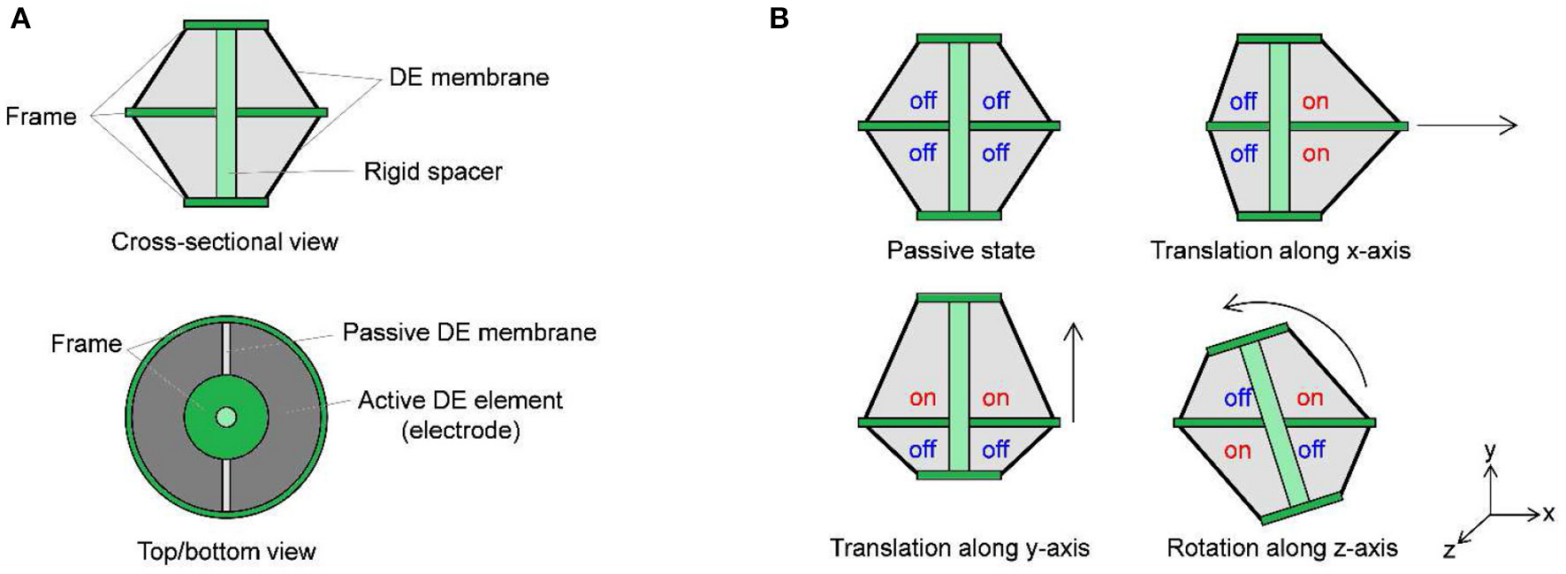
Principle of MDOF motion creation with segmented DE membrane: system cross-sectional and top/bottom views **(A)**, and possible actuation modes **(B)**.

While most of current DE actuator applications operate by performing a one-dimensional stroke only, robotic systems usually require a multi-degree of freedom (MDOF) motion. Different possibilities to realize this motion via DE transducers have been presented in the literature. One common approach is based on converting the in-plane elongation of rolled DEAs into complex motions via the actuator design. In this way, it is possible to move fingers (Jung et al., 2006), legs (Nguyen et al., 2014b), or wings (Lau et al., 2014) of soft robots. Another option consists of exploiting the bending motion of DEAs, which can be realized with rolled actuators (Pei et al., 2004a,b) as well as planar ones (Kofod et al., 2007; Shian et al., 2015a,b). Due to the flexibility and adaptability of DE material, also complex types of bio-inspired motion can be realized, e.g., the swimming of a jellyfish (Godaba et al., 2016) or crawling of worms (Jung et al., 2007). As an alternative way, a MDOF actuation can be generated by patterning several electrodes on a DE membrane (e.g., via screen printing), in such a way that each one of them can be activated individually. Among the different types of MDOF DE configurations presented in the literature, the so-called double cone DEA (DC-DEA) design has been adopted by a number of authors. More specifically, a DC-DEA consists of two circular out-of-plane DEAs pre-stressed against each other. By dividing the electrode of one membrane into two or more segments, the axis of the actuator can be moved in many different ways. An example of DC-DEA with electrodes partitioned in two segments is shown in Figure 2. Depending on which combination of electrode segments is activated, vertical, horizontal, or rotational motion can be achieved, see Figure 2B. A VHB based actuator with four segments on each membrane is presented by Conn and Rossiter (2012). Stroke along different directions, force and moment outputs, as well as the frequency response are characterized by the authors. Choi et al. (2003) designed a MDOF actuator consisting of two DE membranes with four segments each, and illustrated the potential of the DC-DEA for soft robotic systems by building a hexapod robot out of several of similar actuators (Nguyen et al., 2014a). A similar principle is also used by Branz and Francesconi (2016) for developing a DC-DEA with two membranes, segmented into two parts. A finite element model of the system is also developed and experimentally validated. In Cao and Conn (2018), an analytical model has been presented to describe the deformation profile of a DC-DEA. By means of the above model, the authors investigated the effects of geometry and material pre-stretch on the actuator stroke and work output. A dynamic model of a double cone DE vibrissal system, based on combining hyperelasticity theory and Euler-Bernoulli beam equations, is presented in Conn et al. (2012). The model is shown to predict experimental results of the given system in terms of both stroke and deflection.

From the above examples, it can be concluded that many types of DC-DEAs have currently been developed for different applications, each one designed with different materials, configurations, and geometrical parameters. However, there is still a lack of systematic studies on how the different design parameters determine the actuator behavior, e.g., in terms of motion or force range. We remark that, while modeling and design are generally well-understood for simple (i.e., single degree of freedom) DE systems, up to now the literature on MDOF DE actuators lacks systematic experimental studies as well as accurate analytical modeling tools. Once better knowledge on system modeling and characterization is made available, it will allow for better design, optimization, and control of future soft robotics applications, i.e., a tentacle arm which uses many DC-DEAs as modular elements.

The aim of this paper is to understand the relationship between DC-DEA geometry and resulting actuation performance. The overall goal of our research is to use DC-DEAs as constitutive modules for a soft tentacle arm robots. Since the rotational degree of freedom represents the most relevant actuation mode for such application, the relationship between geometry and rotation performance must be properly understood first. Following this goal, in this work we focus on modeling and design optimization of the DC-DEA rotational degree of freedom. To systematically study the effects of geometry on actuator performance, different prototypes are manufactured by changing the inner radius of the membrane as well as the length of the spacer between the two membranes. For each one of them, the maximum rotation angle is examined. The collected data are subsequently used to develop a dynamic model of the soft robotic structure. The presented model shares some similarities with to the one in Conn et al. (2012). However, to obtain a systematic description of the structure non-linearities and electro-mechanical coupling in a control-oriented fashion, a different approach based on the Euler-Lagrange framework is pursued in our work. A control-oriented model is preferred over a finite element one, since it allows for fast parameter identification and numerical simulations. Furthermore, it also provides a framework for the development of future control algorithms. The developed model is then calibrated and used to perform a theoretical parametric study. Based on the results of the numerical simulations, an optimized prototype with improved rotation angle is manufactured. These investigations will provide a basic understanding of the actuator element that is necessary for the development of future robot arms.

The remainder of this paper is organized as follows. In section Design of the MDOF DE Actuation Module, the design of the DE membranes as well as of the overall MDOF actuator is described. In section System Modeling, the simulation model is presented. The experimental setup is presented in section Experimental Characterization and Model Validation, together with the results of the angle measurement and the validation of the simulation model. After that, a parameter simulation is used to optimize the DEAs geometry in terms of the maximum rotation angle, and the performance of the resulting prototype are evaluated in section Actuator Optimization. Finally, section Conclusions provides a final discussion and discusses possible future research directions of the presented work.

## Design of the MDOF DE Actuation Module

**Figure 3:**
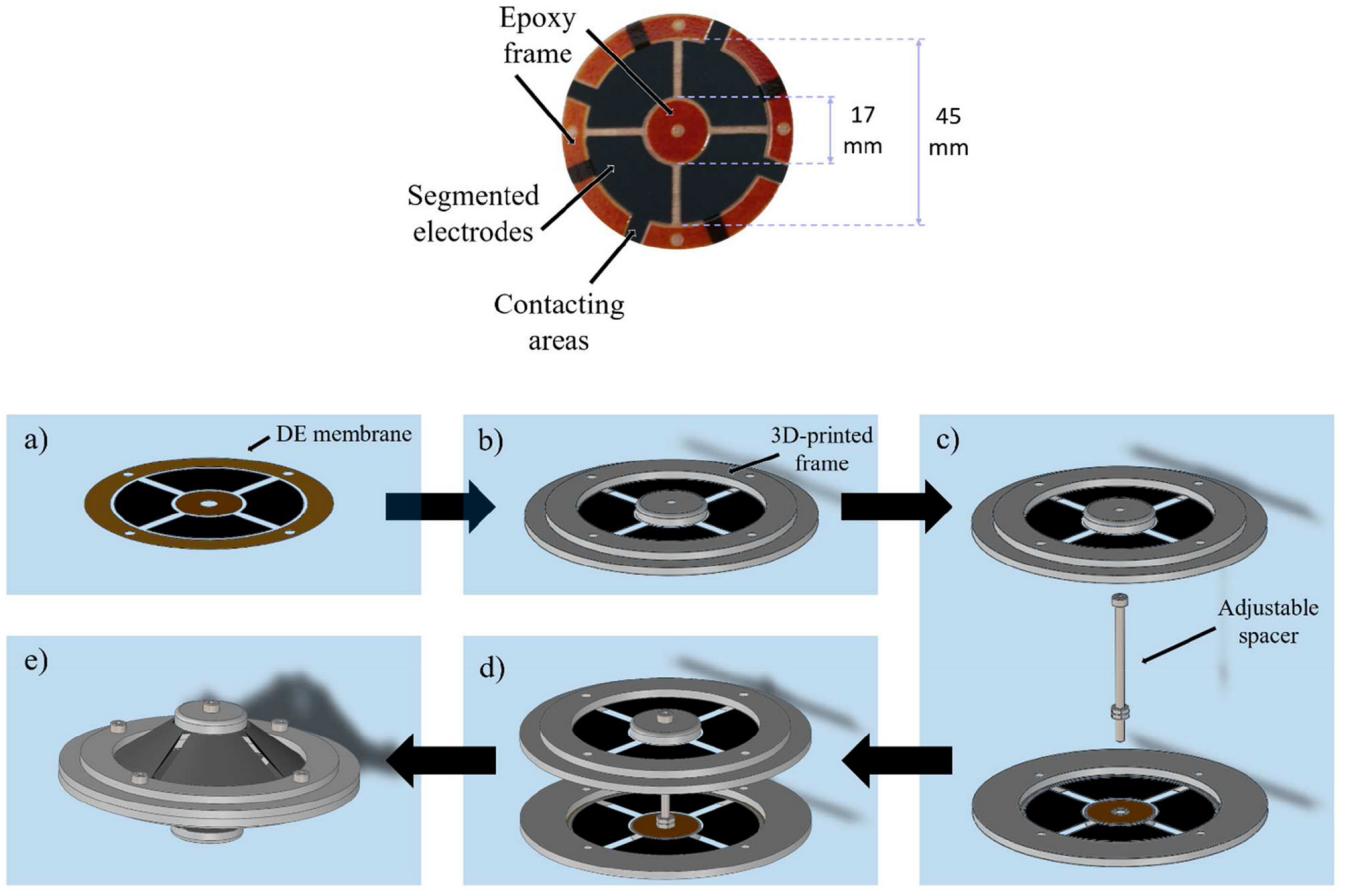
Upper part: picture of the screen printed DE membrane with epoxy frame (orange parts). Lower part: mounting of the DC-DEA, DE membrane **(a)**, adding of 3D-printed frame **(b)**, placing of rigid spacer **(c)**, connecting at inner actuator part **(d)**, and outer actuator diameter **(e)**.

**Figure 4:**
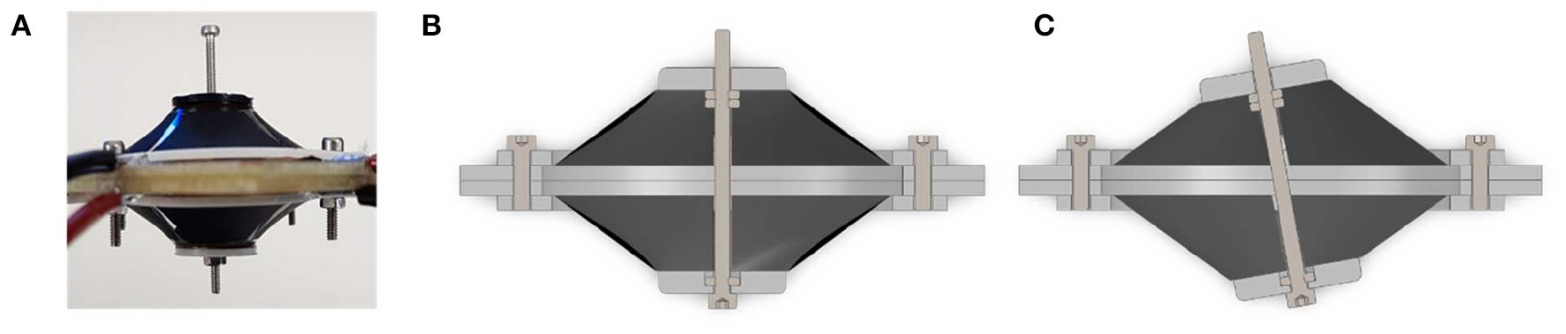
Picture of assembled actuator with *r*_i_ = 8.5 mm and adjustable start deflection *d* **(A)**, CAD section view of actuator assembly without exitation voltage **(B)**, and with excitating the opposite membranes to create a rotational motion **(C)**.

To generate the desired MDOF actuation, a DC-DEA is chosen in this work. The system consists of two circular out-of-plane DE membranes pre-stressed against each other via a rigid spacer. A picture of the adopted circular DE membrane is shown in Figure 3, upper part. For the considered design, outer and inner diameters are equal to 45 and 17 mm, respectively. Carbon black electrodes are screen-printed on a 50 μm silicone membrane (Wacker Elastosil 2030), bi-axially pre-stretched by 10%. The active electrode area of each DE membrane is divided into four equal segments, which can be activated independently. To allow the membrane to deform out-of-plane, an epoxy frame is applied at both outer and inner circumferences (orange parts in Figure 4). It is pointed out that the overall membrane design is similar to other actuators presented in literature (e.g., Choi et al., 2003; Conn and Rossiter, 2012; Branz and Francesconi, 2016). Differently from the mentioned works, however, the DE material adopted in our paper is silicone, rather than VHB. As a result, it is expected that it provides different performance in terms of maximum stretch and viscoelastic losses.

To assemble the whole system, 3D-printed circular frames are added at both outer and inner diameters of the membrane (lower part of Figures 3a,b). After that, a rigid spacer is implemented by means of a screw placed between the actuator halves (Figure 3c), and then the two inner parts of both membranes are rigidly connected via the spacer (Figure 3d). Finally, the two outer diameters are joined together with a rigid ring element which, in turn, is connected to a fixed frame (Figure 3e). Due to the flexibility of the DE membranes, the rigid spacer is allowed to move with respect to the rigid outer frame (see Figure 4). Figure 4 shows also the rigid connector in details, and also highlights the screw which allows to modify the spacer length.

When pre-strained out of plane by the rigid spacer, each DE membrane is subject to a radial pre-loading force which depends on the system geometry (i.e., inner diameter, outer diameter, rigid spacer length), as well as by the type of DE material. For the considered cone DE geometries, the existence of such a pre-load is fundamental for the generation of an actuation stroke. Without electrical activation, and provided that gravitational effects are negligible compared to DE elasticity (due to the lightweight structure), the pre-load forces induced by each segment onto the rigid spacer are approximately the same. As a result, at equilibrium the rigid spacer rests in the middle of the two membranes, directed orthogonally to the plane containing the outer rigid frames. By applying high voltage to an electrode segment, the corresponding membrane portion tends to increase its surface as a consequence of the Maxwell stress. This effect creates a mismatch between pre-loading forces, which results into the rigid spacer moving to a new equilibrium configuration. Depending on which combinations of DE segments are activated simultaneously, different actuation modes can be achieved, similarly to Figure 2B. Note, however, that in this case the existence of eight independent actuation variables (four for each membrane) makes it possible to control 5 degrees of freedom (translation along three axes and rotation along two axes) of the rigid spacer, allowing to also achieve more complex motion patterns than simple in-plane translation and rotation.

## System Modeling

**Figure 5:**
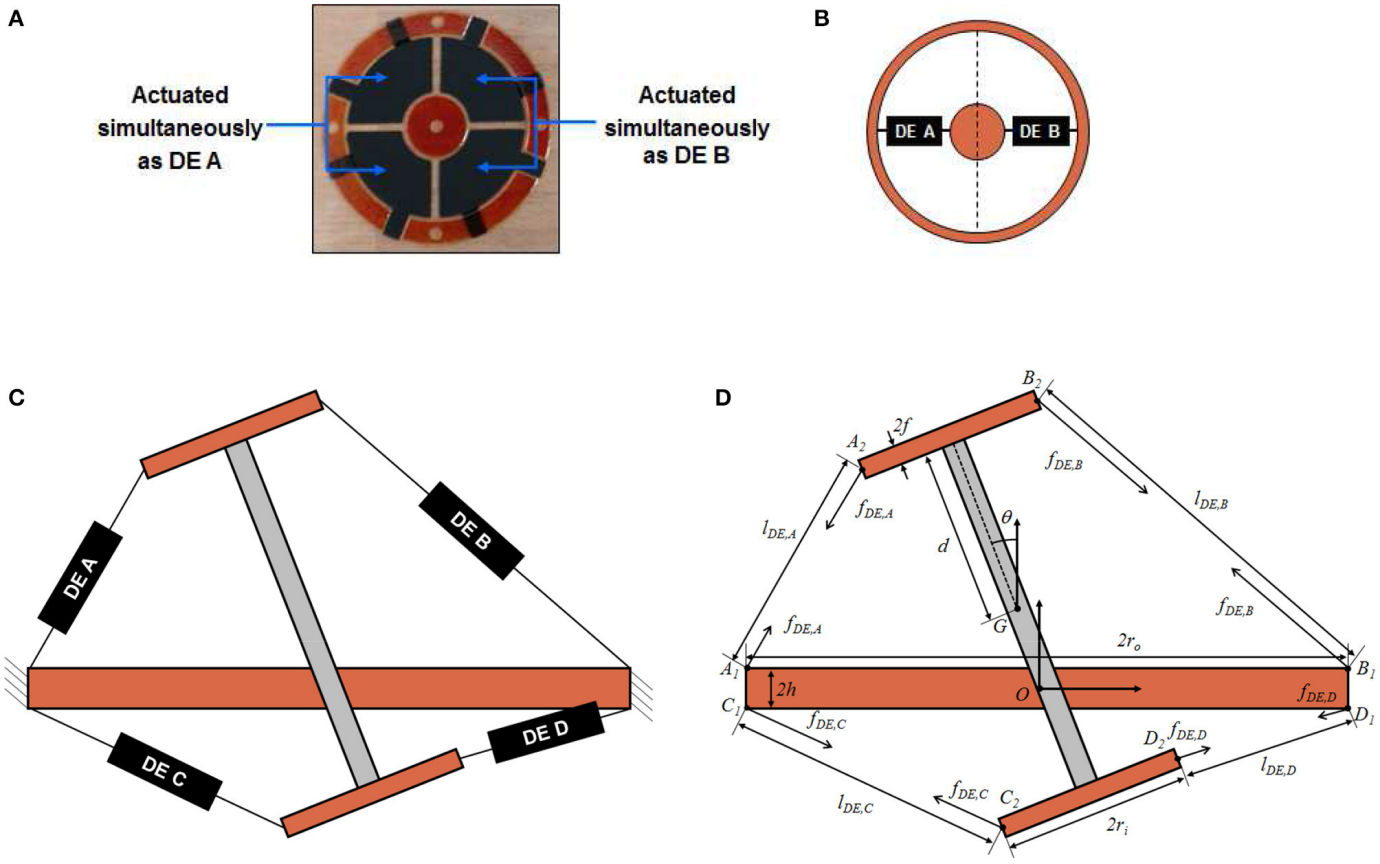
Circular DE membrane activation for in-plane rotation, upper view **(A)**, and equivalent lumped representation **(B)**. Cross-sectional sketch of DC-DEA **(C)**, and equivalent structure model which highlights all geometrical parameters **(D)**.

To better understand the relationship between system geometry and actuation performance, a control-oriented model of the DC-DEA is developed in this section. Since in this work we primarily focus on the in-plane rotation angle, the model is restricted to the in-plane motion, implying that only four independent actuation variables exist (corresponding to pairs of adjacent electrode segments). To this end, we consider the sketch of the DC-DEA shown in Figure 5A. Each pair of adjacent DE segments is represented by an equivalent lumped DE element, placed in the middle of the corresponding membrane segment (see Figure 5B). Based on this equivalent representation of the DE membranes, a cross-sectional view of the complete DC-DEA is shown in Figure 5C. Note that four equivalent membranes appear in this picture, labeled as DE A (upper-left), DE B (upper-right), DE C (lower-left), and DE D (lower-right), respectively.

It is assumed that the deformation state of each membrane can be completely described by the distance between the points connecting the equivalent DE element and the rigid parts of the structure (i.e., inner circle and outer frame in Figure 5B). As a result, the force provided by each DE is assumed to be a function of the distance between those points, as well as the applied voltage. We point out that this kind of simplifying assumption is common when modeling single-DOF cone DE membrane in a control-oriented fashion (Rizzello et al., 2015). This modeling choice is therefore needed in order to treat each DE membrane as a lumped element.

Based on the above considerations, each DE element can be replaced with an equivalent force, acting along the line connecting the membrane attachment points. A sketch of the resulting system is then shown in Figure 5D. The relevant geometric quantities appearing in Figure 5D are listed in the following:

- *r*_*i*_: DE membrane inner radius;
- *r*_*o*_: DE membrane outer radius;
- *d*: half length of the rigid spacer;
- *h*: half thickness of the frame connecting the two membranes;
- *f* : half thickness of the frame connecting the rigid spacer to the inner frame of each membrane;
- *O*: origin of a fixed right-handed reference frame, attached to the geometric center of the frame connecting the two membranes, and oriented such that the *y*-axis is orthogonal to the ground;
- *K*_1_ = [*K*_1*x*_ *K*_1*y*_]^*T*^, *K* ∈ {*A, B, C, D*}: position of attachment point between DE membrane *K* and outer frame connected to a fixed reference, measured with respect to the frame having origin in *O*;
- *K*_2_ = [*K*_2*x*_ *K*_2*y*_]^*T*^, *K* ∈ {*A, B, C, D*}: position attachment point between DE membrane *K* and inner frame connected to the rigid spacer, measured with respect to the frame having origin in *O*;
- *G* = [*G*_*x*_ *G*_*y*_]^*T*^: position of the center of mass of the rigid spacer, measured with respect to the frame having origin in O;
- θ: rotation angle of the rigid spacer with respect to its center of mass;
- *l*_*DE, K*_, *K* ∈ {*A, B, C, D*}: equivalent length of DE membrane *K*.

Based on the above discussion, DE membranes and structure can be treated as two interconnected systems. In the following, independent models are developed for both structure and DEs. Afterwards, a proper interconnection among those parts if performed, leading to an electro-mechanically coupled model of the complete system.

### Structure Model

To model the structure, a Lagrangian approach is pursued in this work (Goldstein et al., 2002). The goal is to develop a general modeling framework which can be used for both system analysis, simulation, and control. Note that the only moving part of the system is the rigid spacer, which can be considered as an unconstrained rigid body. For the in-plane motion case, only three independent variables are necessary to describe the system configuration, which are conveniently chosen as *G*_*x*_, *G*_*y*_, and θ. The overall system Lagrangian, denoted by L, is given as a function of the independent variables as follows

(2) $$\begin{array}{l} L=\frac{1}{2}m\left({{\dot{G}}_{x}}^{\;2}+{{\dot{G}}_{y}}^{\;2}\right)+\frac{1}{2}I{\overset{∙}{\theta }}^{2}-mgG_{y}. \end{array}$$

The terms appearing on the right-hand side of (2) represent the translational kinetic energy of the spacer, the rotational kinetic energy of the spacer, and the negated gravitational potential energy of the spacer, respectively. Quantities *m* and *I* resent the rigid spacer mass and moment of inertia with respect to the center of mass, respectively. Based on (2), the equations of motion are given as follows

(3) $$\begin{array}{l} \frac{d}{dt}\frac{\partial L}{\partial {\dot{G}}_{x}}-\frac{\partial L}{\partial G_{x}}=\tau_{Gx}\;\to \;m{\ddot{G}}_{x}=\tau_{Gx}, \end{array}$$

(4) $$\begin{array}{l} \frac{d}{dt}\frac{\partial L}{\partial {\dot{G}}_{y}}-\frac{\partial L}{\partial G_{y}}=\tau_{Gy}\;\to \;m{\ddot{G}}_{y}+mg=\tau_{Gy}, \end{array}$$

(5) $$\begin{array}{l} \frac{d}{dt}\frac{\partial L}{\partial \overset{∙}{\theta }}-\frac{\partial L}{\partial \theta }=\tau_{\theta }\;\to \;I\ddot{\theta }=\tau_{\theta }, \end{array}$$

where τ_*Gx*_, τ_*Gy*_, and τ_θ_ represents the generalized forces. Note that, while *G*_*x*_ and θ are only affected by the corresponding generalized forces, an additional contribution due to gravity can be observed in the equation describing the dynamics of *G*_*y*_.

### DE Membranes Model

The DE membranes model relates the force of each DE, denoted as *f*_*DE, K*_ in Figure 5D, to the corresponding membranes lengths *l*_*DE, K*_ and applied voltage *v*_*DE, K*_, for *K* ∈ {*A, B, C, D*}. In principle, modeling of the DE behavior is challenging due to the large deformation, strong non-linearities, and rate-dependent effects exhibited by the material. To keep the mathematical complexity of the model sufficiently simple, we assume that the force of membrane *K* only depends on deformation and voltage of the same membrane, thus neglecting neighboring effects among different segments of the same DE. As a result, the overall model of the four DE membranes can be expressed as four independent models in the following form

(6) $$\begin{array}{l} f_{DE,K}=f_{DE,K}\left(l_{DE,K},v_{DE,K}\right),\;K\in \left\{A,B,C,D\right\}. \end{array}$$

By using the results in Rizzello et al. (2015), DE length *l*_*DE, K*_, force *f*_*DE, K*_, and voltage *v*_*DE, K*_ can be normalized into radial stretch λ_*DE, K*_, true radial stress σ_*DE, K*_, and true electric field *E*_*DE, K*_, as follows

(7) $$\begin{array}{l} \lambda_{DE,K}=\frac{l_{DE,K}}{r_{o}-r_{i}}, \end{array}$$

(8) $$\begin{array}{l} \sigma_{DE,K}=\frac{2\lambda_{DE,K}}{\pi z_{0}\left(r_{0}^{2}-r_{i}^{2}\right)}f_{DE,K}, \end{array}$$

(9) $$\begin{array}{l} E_{DE,K}=\frac{\lambda_{DE,K}}{z_{0}}v_{DE,K}, \end{array}$$

where *z*_0_ is the thickness of the cone DE membrane in undeformed configuration. Since the model description is based on true radial stress and true electric field, rather than on nominal ones, it allows to predict how those quantities change according to the current membrane geometry and deformation. Once these quantities are available, a constitutive DE material model can be expressed, in first approximation, as follows (Rizzello et al., 2015)

(10) $$\begin{array}{l} \sigma_{DE,K}=\overset{N}{\underset{i=1}{\sum }}\left(\beta_{i}\lambda_{DE,K}^{\alpha_{i}}-\gamma_{i}\lambda_{DE,K}^{-\alpha_{i}}\right)-\varepsilon_{0}\varepsilon_{r}E_{DE,K}^{2}+\eta_{v0}{\overset{∙}{\lambda }}_{DE,K}, \end{array}$$

where α_*i*_, β_*i*_, γ_*i*_, *i* = 1,…, *N*, are constitutive parameters describing a modified *N*-th order Ogden model, and η_*v*0_ represents a viscoelastic damping coefficient. Clearly, the same material parameters will be used to describe each one of the four membranes. Note that Equation (10) is described in terms of radial stretch only, since for cone DEAs undergoing homogeneous deformations it is typically to assume that a constant circumferential stretch (Rizzello et al., 2015) (pure-shear assumption).

### Overall System Model

Independent models for both structure and DE membranes were developed in the previous section. On the one hand, the structure model expressed by Equations (3)–(5) receives generalized forces τ as input, and returns Lagrangian parameters *q* as outputs, where

(11) $$\begin{array}{l} q={\left[\begin{array}{c} G_{x}G_{y}\theta \end{array}\right]}^{T}, \end{array}$$

(12) $$\begin{array}{l} \tau ={\left[\begin{array}{c} \tau_{Gx}\tau_{Gy}\tau_{\theta } \end{array}\right]}^{T}. \end{array}$$

On the other hand, the DE model given by Equations (6)–(10) makes use of DE lengths *l*_*DE*_ and DE voltages *v*_*DE*_ to compute the DE forces *f*_*DE*_, where

(13) $$\begin{array}{l} l_{DE}={\left[\begin{array}{c} l_{DE,A}l_{DE,B}l_{DE,C}l_{DE,D} \end{array}\right]}^{T}, \end{array}$$

(14) $$\begin{array}{l} v_{DE}={\left[\begin{array}{c} v_{DE,A}v_{DE,B}v_{DE,C}v_{DE,D} \end{array}\right]}^{T}, \end{array}$$

(15) $$\begin{array}{l} f_{DE}={\left[\begin{array}{c} f_{DE,A}f_{DE,B}f_{DE,C}f_{DE,D} \end{array}\right]}^{T}. \end{array}$$

Since both models are expressed in terms of a different set of force-displacement coordinates, a direct interconnection between the two is not possible. However, suitable coordinate transformations can be introduced to relate θ to *l*_*DE*_ and *f*_*DE*_ to τ, respectively, so that a consistent coupling among the two subsystems can be effectively achieved.

As a first step, we determine the relationship between θ and *l*_*DE*_ by means of a kinematic model. Based on Figure 5D, the coordinates of points *A*_1_, *A*_2_, *B*_1_, *B*_2_, *C*_1_, *C*_2_, *D*_1_, *D*_2_ with respect to fixed origin *O* can be computed as functions of *q*, as follows:

(16) $$\begin{array}{l} A_{1}=\left[\begin{array}{c} -r_{o}\\ h \end{array}\right], \end{array}$$

(17) $$\begin{array}{l} A_{2}\left(q\right)=\left[\begin{array}{c} G_{x}-\left(d+f\right)\sin\theta -r_{i}\cos\theta \\ G_{y}+\left(d+f\right)\cos\theta -r_{i}\sin\theta \end{array}\right], \end{array}$$

(18) $$\begin{array}{l} B_{1}=\left[\begin{array}{c} r_{o}\\ h \end{array}\right], \end{array}$$

(19) $$\begin{array}{l} B_{2}\left(q\right)=\left[\begin{array}{c} G_{x}-\left(d+f\right)\sin\theta +r_{i}\cos\theta \\ G_{y}+\left(d+f\right)\cos\theta +r_{i}\sin\theta \end{array}\right], \end{array}$$

(20) $$\begin{array}{l} C_{1}=\left[\begin{array}{c} -r_{o}\\ -h \end{array}\right], \end{array}$$

(21) $$\begin{array}{l} C_{2}\left(q\right)=\left[\begin{array}{c} G_{x}+\left(d+f\right)\sin\theta -r_{i}\cos\theta \\ G_{y}-\left(d+f\right)\cos\theta -r_{i}\sin\theta \end{array}\right], \end{array}$$

(22) $$\begin{array}{l} D_{1}=\left[\begin{array}{c} r_{o}\\ -h \end{array}\right], \end{array}$$

(23) $$\begin{array}{l} D_{2}\left(q\right)=\left[\begin{array}{c} G_{x}+\left(d+f\right)\sin\theta +r_{i}\cos\theta \\ G_{y}-\left(d+f\right)\cos\theta +r_{i}\sin\theta \end{array}\right]. \end{array}$$

The overall kinematic model is then given as follows

(24) $$\begin{array}{l} l_{DE}\left(q\right)=\left[\begin{array}{c} l_{DE,A}\left(q\right)\\ l_{DE,B}\left(q\right)\\ l_{DE,C}\left(q\right)\\ l_{DE,D}\left(q\right) \end{array}\right]=\left[\begin{array}{c} \left\|A_{2}\left(q\right)-A_{1}\right\|\\ \left\|B_{2}\left(q\right)-B_{1}\right\|\\ \left\|C_{2}\left(q\right)-C_{1}\right\|\\ \left\|D_{2}\left(q\right)-D_{1}\right\| \end{array}\right], \end{array}$$

where ||*P*_2_ – *P*_1_|| denotes the Euclidean distance between points *P*_2_ and *P*_1_. The complete expression for Equation (24) is rather complex, and thus it is omitted for conciseness.

Once the kinematic model in Equation (24) is available, the principle of virtual works can be used to relate DE forces to generalized forces in an energy consistent way. In particular, since the structure is assumed to be a conservative system, the principle of virtual works implies that

(25) $$\begin{array}{l} f_{DE}^{T}\delta l_{DE}+\tau^{T}\delta q=0. \end{array}$$

By using Equation (24), and by defining the Jacobian as a 4 × 3 matrix function as follows

(26) $$\begin{array}{l} J\left(q\right)=\frac{\partial l_{DE}\left(q\right)}{\partial q}=\left[\begin{array}{c} \frac{\partial l_{DE,A}\left(q\right)}{\partial G_{x}}\frac{\partial l_{DE,A}\left(q\right)}{\partial G_{y}}\frac{\partial l_{DE,A}\left(q\right)}{\partial \theta }\\ \frac{\partial l_{DE,B}\left(q\right)}{\partial G_{x}}\frac{\partial l_{DE,B}\left(q\right)}{\partial G_{y}}\frac{\partial l_{DE,B}\left(q\right)}{\partial \theta }\\ \frac{\partial l_{DE,C}\left(q\right)}{\partial G_{x}}\frac{\partial l_{DE,C}\left(q\right)}{\partial G_{y}}\frac{\partial l_{DE,C}\left(q\right)}{\partial \theta }\\ \frac{\partial l_{DE,D}\left(q\right)}{\partial G_{x}}\frac{\partial l_{DE,D}\left(q\right)}{\partial G_{y}}\frac{\partial l_{DE,D}\left(q\right)}{\partial \theta } \end{array}\right], \end{array}$$

Equation (25) can be rewritten as

(27) $$\begin{array}{l} \left(f_{DE}^{T}J\left(q\right)+\tau^{T}\right)\delta q=0. \end{array}$$

To let (27) hold for any independent change of *q*, the following must be true

(28) $$\begin{array}{l} \tau =-J^{T}\left(q\right)f_{DE}. \end{array}$$

Equation (28) represents the desired transformation equation.

**Figure 6:**
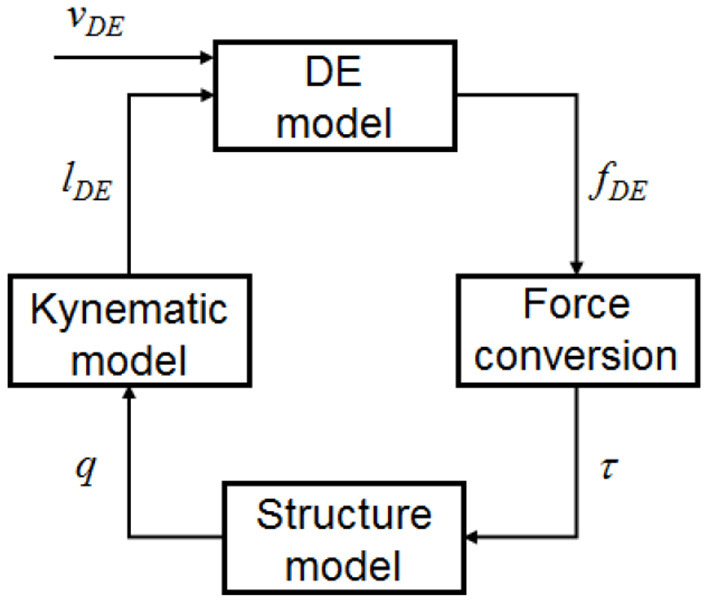
Conceptual block diagram representation of the complete DC-DEA model.

A causal representation of the complete model can be obtained by combining Equations (3)–(10), (24), (26), and (28). The resulting model considers *v*_*DE*_ as independent input, and permits to compute *q* (as well as any other internal variable) as resulting outputs. A conceptual block diagram representation of the overall model, including corresponding coupling terms and transformation equations, is provided in Figure 6. For concluding this section, we point out that the DE model in Equation (10) requires not only *l*_*DE*_ as mechanical input, but also its time derivative (due to the presence of the stretch rate term). Despite this issue, a causal interconnection between the two dynamic blocks can still be obtained if we consider that

(29) $$\begin{array}{l} {\overset{∙}{l}}_{DE}\left(q,\overset{∙}{q}\right)=J\left(q\right)\overset{∙}{q}, \end{array}$$

and that both *q* and its time derivative are outputs of the structure model. Performing a velocity dependent interconnection based on Equation (29) is straightforward, and does not violate causality during the implementation. Nevertheless, this additional coupling term is not reported explicitly in Figure 6, since this figure is intended to represent a conceptual scheme rather than an accurate simulation block diagram.

## Experimental Characterization and Model Validation

Experimental characterization of the prototype is performed in this section. Note that the actuator possess many degree of freedom, therefore the characterization of its performance can be achieved in many different ways. For this work we choose to investigate and to validate the simulation model based on the rotational motion, which is described by the rotation angle θ. Since the maximum rotation angle of a single DC-DEA is an important parameter for determining the overall bending of a tentacle arms, it is chosen as the main quantity to be investigated in this work. Data on rotation angle are collected for different geometries, and are subsequently used to calibrate and validate the model developed in section System Modeling.

### Experimental Setup and Test Procedure

**Figure 7:**
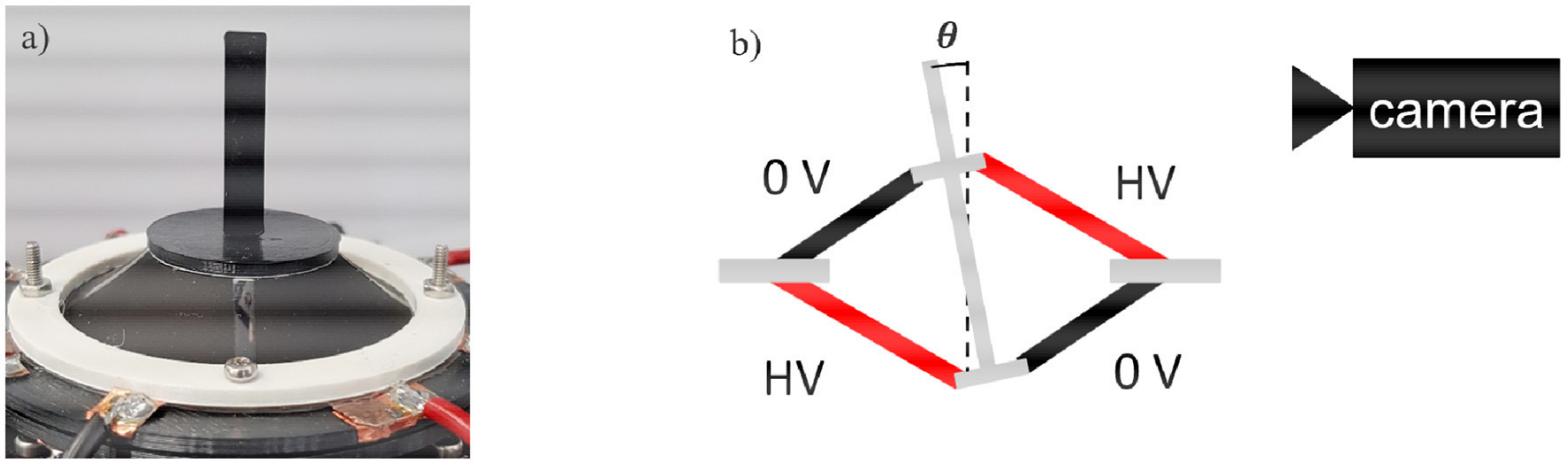
Picture **(a)** and schematic structure **(b)** of the experimental setup for the characterization of the DC-DEA rotation angle.

The aim of this first investigations is the analysis of the achievable rotation angle. A LabView script is used to record a video of the moving actuator as well as to control the actuation. With the NI USB-6343 Multifunction I/O device, a signal between 0 and 10 V is generated and sent to a precision high voltage amplifier (UltraVolt® HVA series 4HVA24-P1) that scales it up to 4 kV. A low-pass filtered step signal is implemented in LabVIEW to precisely control the speed and amplitude of the actuation. After recording a video of the actuator motion with a camera (Pulnix TMC-1405GE), the rotation angle is measured with the help of the edge detection algorithm implemented in Matlab. To improve the accuracy of the rotation angle measurement, the axis of the spacer actuator is extended with an additional rigid element (see Figure 7).

In order to obtain a rotational motion, the opposite electrodes are activated with the same amount of high voltage, as shown in Figure 7. The non-activated electrodes are always kept at 0 V, to ensure as much symmetry as possible during the rotation.

The excitation signal that is given to the activated membranes is chosen as a step signal, which starts from 0 V and transitions to a high voltage level which depends on the specific geometry. More specifically, to ensure repeatable conditions among the different actuators and also to operate below the breakdown strength of the adopted silicone material, the voltage amplitude is chosen so that when the rigid spacer is not displaced (i.e., *q* = 0) the electric field equals *E*_*DE, Max*_ = 80 V/μm WackerChemie. An estimation of the voltage required to generate a desired electric field, given a specific actuator geometry, can be obtained by combining Equations (7), (9), (16), (17), and (24), i.e.,

(30) $$\begin{array}{l} v_{DE,Max}=\frac{z_{0}}{\sqrt{1+{\left(\frac{d+f-h}{r_{o}-r_{i}}\right)}^{2}}}E_{DE,Max}. \end{array}$$

### Parameter Variation

**Figure 8:**
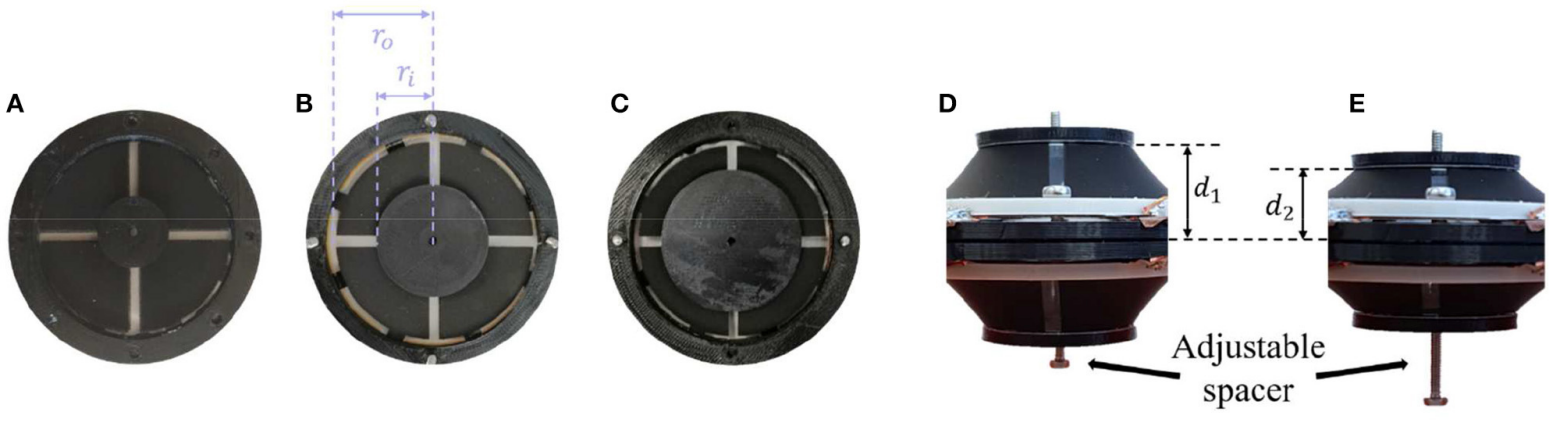
Assembled DE membranes with different inner radii of *r*_i_ = 8.5 mm **(A)**, *r*_i_ = 12.5 mm **(B)**, and *r*_i_ = 16.5 mm **(C)**, and different spacer length d_1_ **(D)** and d_2_ **(E)**.

To study the effect of geometrical parameters on the actuator performance, several prototypes with different values of rigid spacer length and inner radius of the circular DE membrane are designed. The variation of the rigid spacer is implemented by a screw that connects the two rigid inner parts of the membranes (Figure 4). In this way, the value of *d* (i.e., the half of the rigid spacer length) can be varied from 4 to 14 mm with increments of 1 mm. For testing different values of inner radius, three different types of DE membranes are manufactured, having values of *r*_*i*_ of 8.5, 12.5, and 16.5 mm, while *r*_*o*_ is kept constant at 22.5 mm (see Figure 8). Different combinations of *d* and *r*_*i*_ are subsequently tested. The excitation voltage is chosen for each case based on equation (30), with *E*_*DE, Max*_ = 80 V/μm.

**Figure 9:**
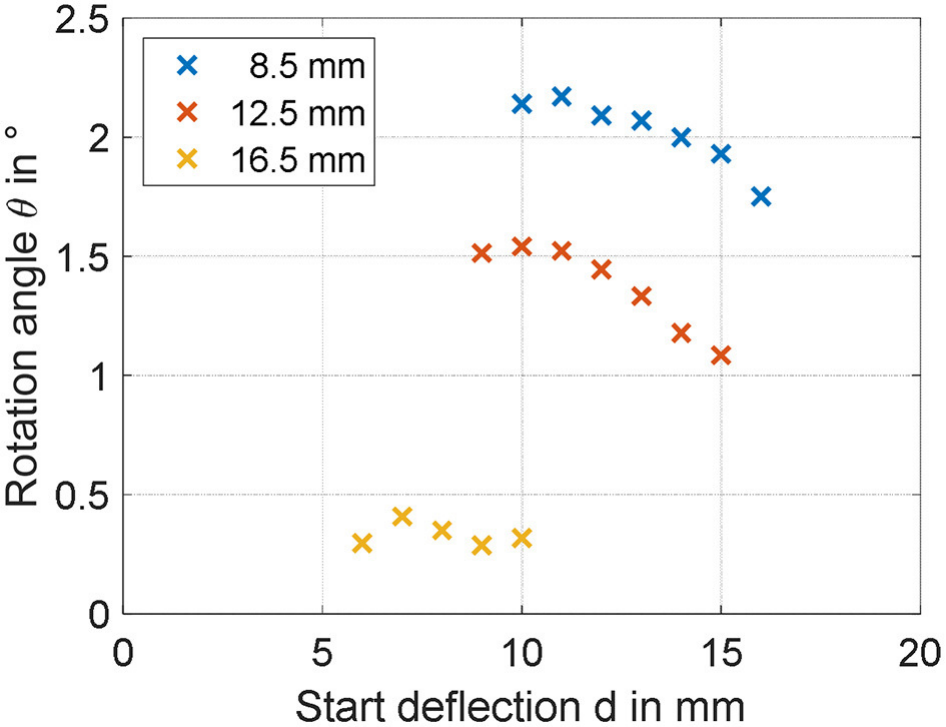
Experimental identification of geometry dependencies on maximum rotation angle for *r*_i_ of 8, 12.5, and 16.5 mm.

The steady-state rotation angles measured for all of the designed actuators are shown in Figure 9. It can be clearly noted that decreasing leads *r*_*i*_ to a larger rotation angle, at least in the investigated range. Concerning the spacer length, a general decrease in angle is observed for increasing values of *d*. The maximum achieved angle among all the manufactured actuators equals 2.2 degrees, and corresponds to *r*_*i*_ = 8.5 mm and *d* = 9 mm.

### Validation of the Simulation Model

**Figure 10:**
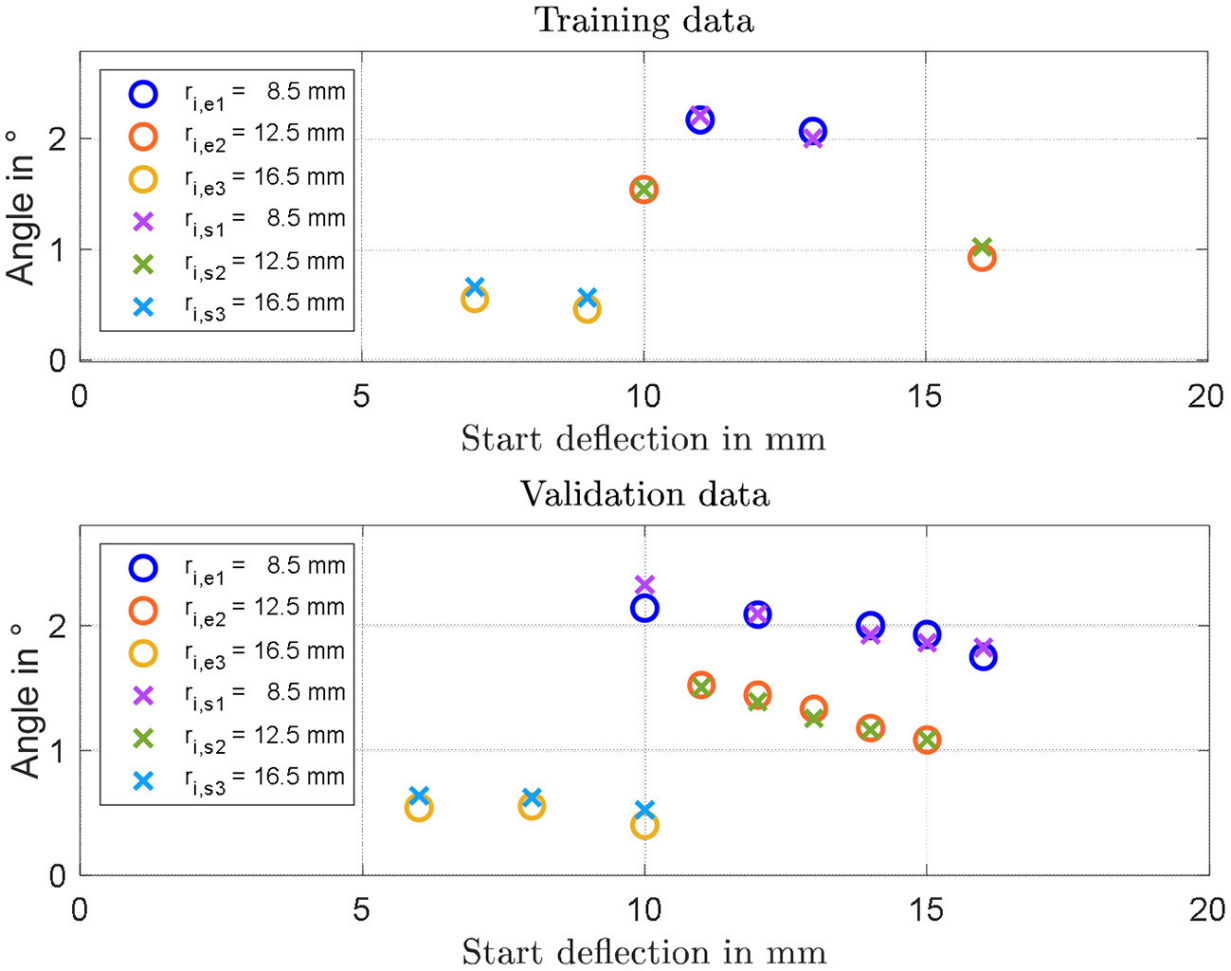
Results of model identification and calibration **(Upper part)**, and experimental validation **(Lower part)**. In each figure, circles represent experiments and crosses represent model predictions.

**Figure 11:**
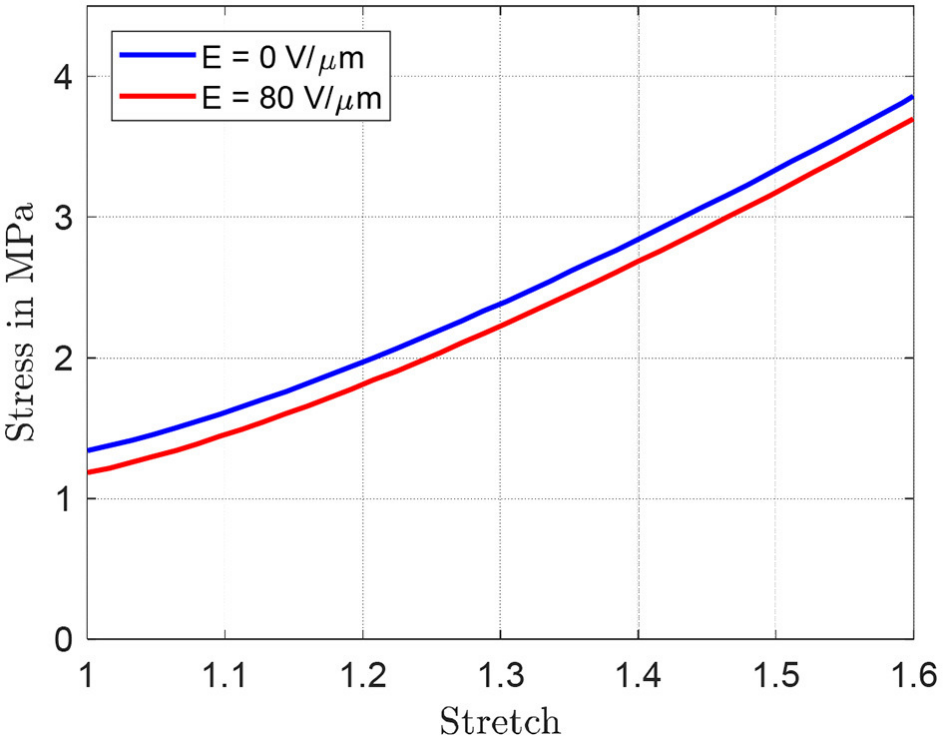
Optimized DE material model.

By inspecting the results in Figure 9, it can be expected that the maximum angle can be increased by reducing both *r*_*i*_ and *d*. To better quantify the effect of both parameters, the simulation model developed in section System Modeling is calibrated based on the collected steady-state measurements. Some of the system parameters can be measured or estimated with sufficiently high accuracy, i.e., the geometrical and inertial ones, and are reported in Table 1. Values of *m* and *I* differ for each experiment, and are not explicitly reported for conciseness. More specifically, among the many experiments, *m* ranges within 2.5 and 9.1 g, while *I* varies within 0.17 and 2.14 kg·mm^2^. The parameters which need to be identified represent the DE constitutive parameters, i.e., α_*i*_, β_*i*_, γ_*i*_, *i* = 1,…, *N*, ε_*r*_, and η_*v*0_. Among those parameters, ε_*r*_ is selected equal to 2.8 according to the material producer datasheet, while the other ones are calibrated based on a non-linear least square method. For identification purpose, the 19 performed experiments, each one corresponding to a different DC-DEA geometry, are split into a training set and a validation set. The training set consists of six randomly selected experiments, 2 for each *r*_*i*_, which are used to calibrate all the unknown model parameters. The result of this calibration is shown in the upper part of Figure 10. After calibration, the remaining 13 experiments are used for the model validation purpose. The results of the validation are shown in Figure 10, lower part. It can be seen that the simulation model well-predicts the rotation angle of the DC-DEA for each of the considered geometries, despite the calibration being performed only with 30% of the collected data. As a result, we conclude that the developed model permits a satisfactory extrapolation of results. In Figure 10, measured data are reported as circles, while calibration (upper part) and validation data (lower part) are represented as crosses. Finally, the stress-stretch curves of the optimized DE material model is shown in Figure 11, for both *E* = 0 V/μm and *E* = 80 V/μm.

**Table 1:** System parameters used for the simulations.

**Parameter**	**Value**	**Unit**	**Status**
*r*_o_	22.5	mm	Known
*h*	2	mm	Known
*f*	0	mm	Known
*g*	9.807	m/s^2^	Known
ε_*0*_	8.854	pF/m	Known
ε_*r*_	2.8	–	Identified
α_*i*_	[2, 4]	–	Identified
β_*i*_	[1.707, −0.023]	MPa	Identified
γ_*i*_,	[1.298, −0.955]	MPa	Identified
η_*v*0_	0.2	MPa·s	Identified

## Actuator optimization

The validation procedure described in the previous section highlighted the ability of the developed model in predicting the steady-state response for different geometrical configurations. This fact will be used in the present section to perform a parameter study which, in turn, will be exploited to optimize the design of the structure for achieving high rotation angle. Torque performance is not considered in here, since in this study we are mostly interested in kinematic optimization of soft tentacle arms.

### Parameter Study

**Figure 12:**
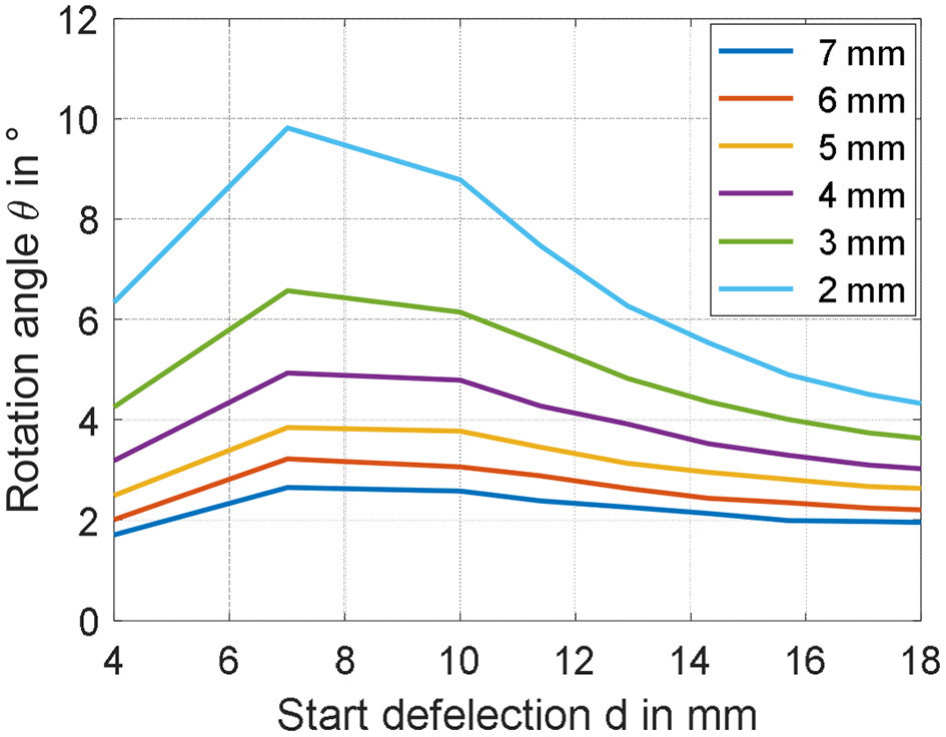
Parameter studies showing the appreciable increase of the rotation angle by decreasing the inner radius *r*_i_ as well as the start deflection *d*.

The simulation model characterized in section Validation of the Simulation Model is here used to study the effect the two investigated parameters. The range of both inner radius and start deflection is extended with respect to the previous measurements, to see for which values of parameters the rotation angle can be increased in a meaningful way. Figure 12 shows the results of the simulation for values of *r*_*i*_ ranging from 2 to 7 mm, and *d* varying between 4 and 18 mm. Since from studies conducted in section Experimental Characterization and Model Validation, it is observed that the value of *m* has a low impact on the steady performance, no gravitational force is considered for this study, making the resulting actuator motion completely symmetric. From Figure 12, it is clearly visible that the rotation angle can be significantly affected by changing the geometry of the actuator. According to the model, a smaller start deflection in combination with a small inner radius increases the rotation angle of the actuator in the range around 10 degrees. Rotational actuator motions in this range allows to significantly improve the effectiveness of the considered actuator for different application scenarios. As a final remark, note that the start deflection defines both the moment lever arm and the pre-stress in the material at the same time. This relationship is highly complex to quantify, due to both structure and DE material non-linearities. It is reasonable to assume that the maximum observable in Figure 12 represents indeed the optimal trade-off between all those effects, which leads to the maximum resulting rotation angle θ.

### Optimized Actuator Design

**Figure 13:**
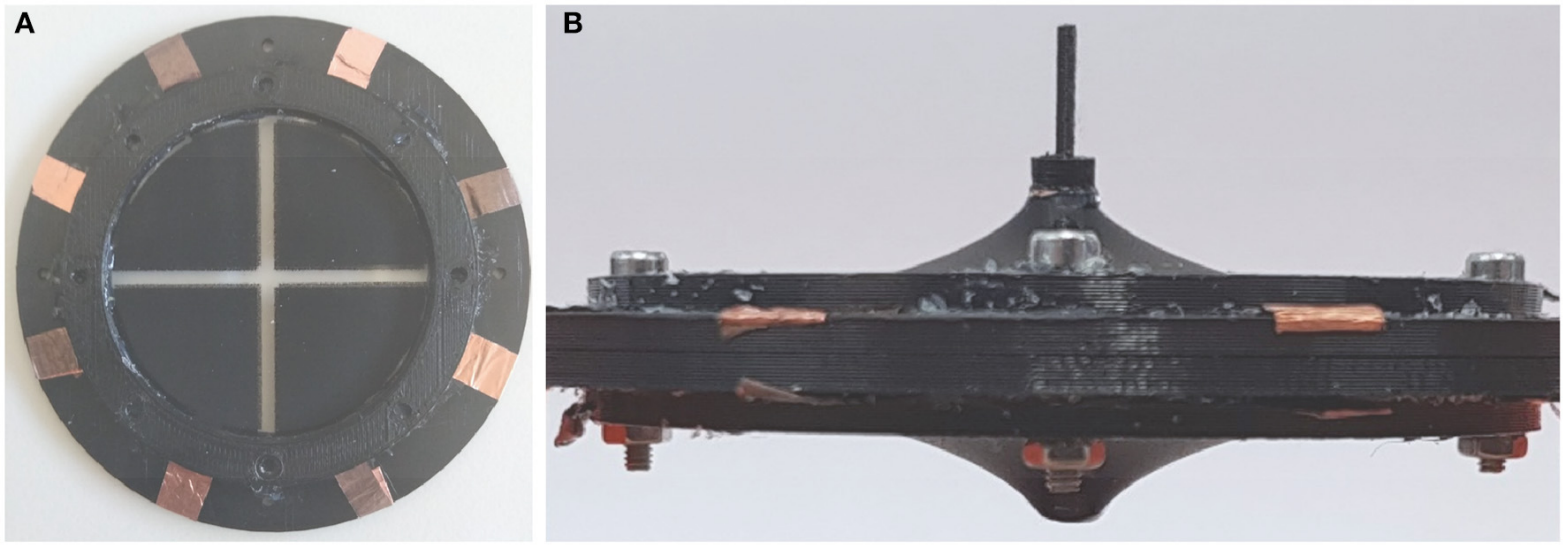
Adapted DE membrane **(A)** and optimized actuator with *r*_i_ = 2 mm and *d* = 10 mm **(B)**.

Based on the results of the previous sections, novel prototypes are realized and tested. According to the results in Figure 12, the rotation angle can be maximized by reducing the inner radius *r*_*i*_ as much as possible, and by setting the spacer parameter *d* = 7 mm. For practical manufacturability, a compromise value of 2 mm is selected for *r*_*i*_. Corresponding to this inner radius, three different values of *d* are tested, i.e., 4 mm (corresponding to the smallest possible spacer length), 7 mm (corresponding to the theoretical maximum angle), and 10 mm (assuming also a value for θ near the maximum). To manufacture membranes having inner radius equal to 2 mm, a novel DE membrane lacking the epoxy frame in the middle is screen-printed (Figure 13A). The resulting DC-DEAs are then assembled according to the required geometries, with one example shown in Figure 13B (i.e., for *d* = 10 mm).

**Figure 14:**
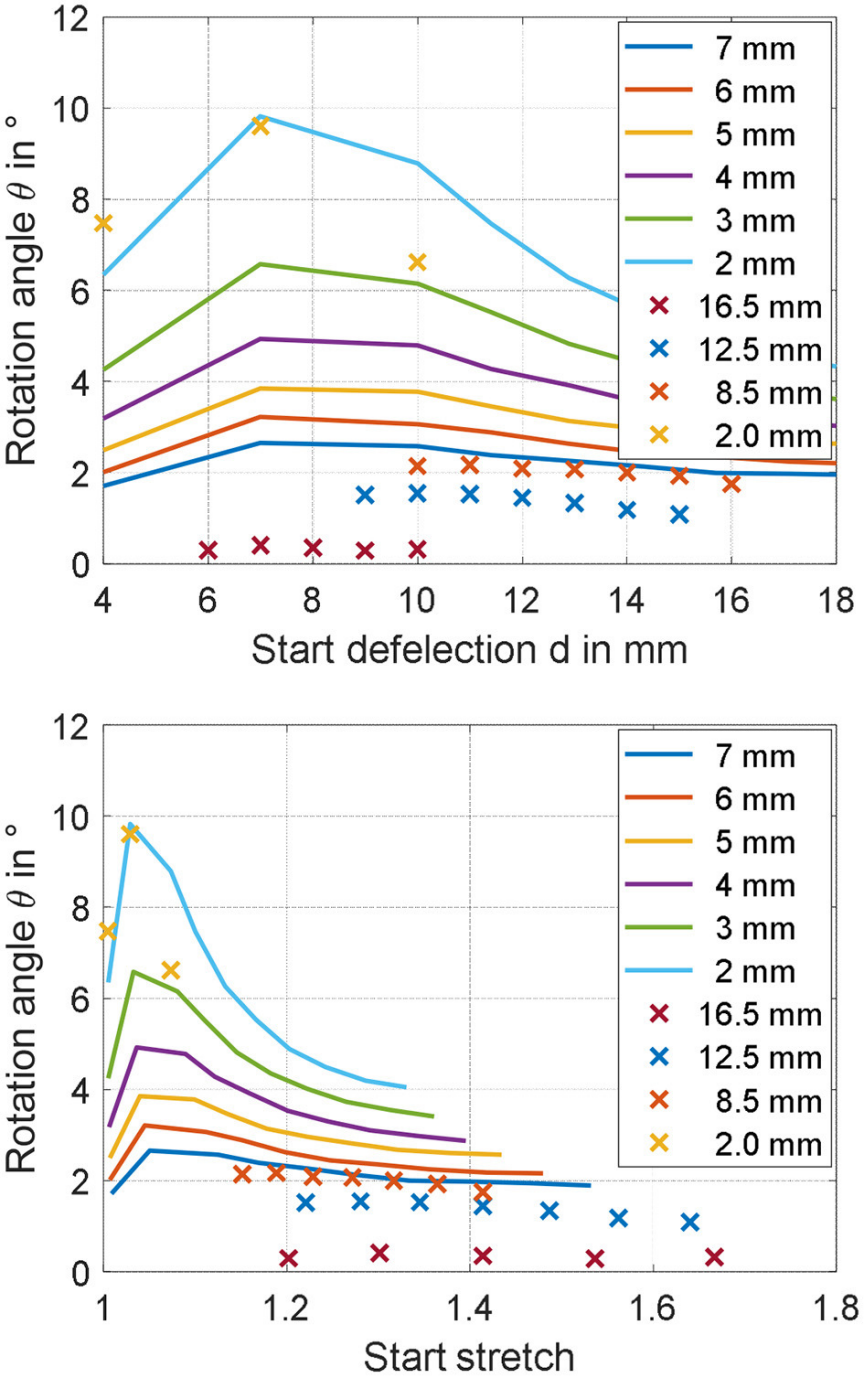
Predicted (continuous lines) and measured (crosses) rotation angles for different *r*_i_, represented as functions of start deflection d **(Upper part)**, and material stretch λ_1_ **(Lower part)**.

Once again, the axis of the rigid spacer is extended to measure the rotation angle generated by the application of a filtered step voltage, given by Equation (30) with *E*_*DE, Max*_ = 80 V/μm. The obtained angles are shown in Figure 14 (upper part), marked as yellow crosses, and equal 7.47 degrees for *d* = 4 mm, 9.60 degrees for *d* = 7 mm, and 6.62 degrees for *d* = 10 mm, respectively. Compared to previous experiments, in which the maximum rotation angle was of 2.2 degrees, a significant improvement can be appreciated. In particular, the 9.60 degrees rotation observed for *d* = 7 mm is sufficiently close to the model prediction, of about 9.80 degrees (error of 2%). Lower, bust still overall satisfactory accuracy is observed for *d* = 4, in which the experimental measurement of 7.47 degrees is sufficiently closed to the simulated angle of 6.35 degrees (error of 15%). For the start deflection of *d* = 10 mm, however, the measured rotation angle of 6.62 deviates from the predicted value of about 8.80 degrees of a more significant amount (error of 25%). The reasons for those inaccuracies may be due to several factors, which are discussed in the following.

First, we point out that the gravity force has been neglected when performing the analysis in section Parameter Study. While it can be safely neglected for the calibration and validation experiments, in which the DE pre-stretch is sufficiently high, this may not be true for the optimized geometries corresponding to barely stretched membranes, i.e., for *d* = 4 mm. Another possible reason for the observed inaccuracy is the unavoidable deviation of the membrane from the ideal cone shape, which is particularly critical for small *r*_*i*_ and large *d*. For the optimized design, this fact is especially true for the two actuators with *d* = 10 mm, and is also clearly visible in Figure 13, where the shape of the membrane is more curved instead of cone-shaped. Despite modeling of such a behavior has already been investigated in the literature, the resulting constitutive relationships are based on spatially-dependent differential equations which appear as unsuitable for our scope (He et al., 2010). Better accuracy could be potentially achieved by employing a more accurate, yet more complex, dynamic model describing the DE material which also accounts for membrane necking in a control-oriented fashion, e.g., an extension of the work in (Rizzello et al., 2018) for cone-shaped geometries. Finally, to understand even more the potential reasons for the observed inaccuracy, both measured and predicted angles are plotted as a function of maximum DE radial stretch λ_1_ in unacted condition, instead of *d*. The maximum DE radial stretch is computed by combining Equations (7) and (16)–(24), and is based on the current geometric parameters *d* and *r*_*i*_ as well as on the maximum simulated angle. The resulting plot is shown in Figure 14, lower part. As it can be observed, the last set of designs lie in a region of low stretch, in which no calibration and validation data are available. As a result, we conclude that despite the model is capable of accurately predict the effect of different geometries in the calibration stretch range, some issues arise when trying to extrapolate the results. It can be foreseen that the improvement of the calibration process, with additional data for lower stretches, can lead to better model predictions.

Despite the issues pointed out above, it can be noted that the error between predicted and measured angles never exceeds 25% for all of the considered optimized designs. Furthermore, it can be observed that the value of the angle is increased of about 4 times compared to the initial designs. As a result, we deduce that performance improvement is still successfully achieved.

## Conclusions

In this work, characterization, modeling, and design optimization of a multi degree-of-freedom dielectric elastomer actuator system for soft robotic applications has been presented. The performed investigation has shown the influence of geometrical scaling on the motion behavior of the DE actuator, expressed in terms of in-plane rotation angle. A control-oriented model has been developed and validated based on the collected data, and has shown a good capability in predicting the achieved angle for different geometrical configurations. The maximum angle achieved with the first set of DC-DEAs is 2.2 degrees, corresponding to the smallest of the three radii (8.5 mm) and a start deflection of 9 mm. Based on the developed model, the effect of geometry on actuation performance have been investigated. As a result of this numerical analysis, an optimized DC-DEA is then assembled. The optimized actuator has been designed with *r*_*i*_ = 2 mm and *d* = 7 mm, and has allowed to achieve a maximum angle of 9.60 degrees with an error between simulations and experiments of 2%. It has been observed, however, that for some of the theoretical optimal geometries the model fails in providing accurate predictions. An error of 15% for *d* = 4 mm, and an error of 25% of *d* = 10 mm, is observed, respectively. This is mostly due to some simplifying assumptions made when developing the model, as well as due to limitation in the data calibration set. Therefore, for future works, more extensive modeling efforts and experimental campaigns will be required for achieving a more accurate scaling prediction. Despite this fact, the first attempt to improve the performance via model-based predictions presented in this paper has led to an overall positive outcome, since the overall maximum angle has been increased from the original value of 2.2 degrees up to 9.60 degrees. As a final remark, we point out that the obtained angle appears smaller than the ones typically obtained by other authors dealing with DC-DEAs, which is typically larger than 10 degrees (usually in the range between 20 and 30 degrees). While most of the authors have used VHB acrylic-based DE materials, in our work we have performed one of the first attempts to develop, model, and optimize a silicone-based DE soft robot. Despite performance comparisons between silicone and VHB materials for DEs are normally conducted for simple actuator configurations, the high non-linearity of the presented structure makes it difficult to quantitatively foresee how such performance difference is reflected into rotational DC-DEA motion. For evaluation purpose, we point out that a comparison between different DE materials for a modified DC-DEA design has been presented by Ghilardi et al. (2017). The amount of rotation angle they obtained with silicone is of about 8 degrees for 3.9 kV, corresponding to 89 V/μm. Such a performance appears as consistent with the results obtained in our experiments. We also point out that, while silicone is characterized by a smaller stretch than other elastomers, it also exhibit many attractive characteristics over VHB which include higher bandwidth, smaller viscoelastic loss, less sensitivity to temperature and humidity, and improved manufacturability. Investigating whether it is possible to achieve motion performance comparable to VHB acrylic DEs while keeping all the advantages of silicone DEs will be one of the major challenges of future research.

Among the next fundamental steps, the design of a DC-DEA module with a larger value of *r*_*o*_ will also be considered. In this way, the resulting actuator will have a smaller ratio between inner and outer radii, thus potentially allowing to further increase the rotation angle even more. Additionally, more complex DEA robotic structures will also be considered for the further optimization of the rotation angle. As an example, the rigid spacer can be replaced by an elastic spacer, like a linear spring or even a bi-stable element. Furthermore, other than the in-plane rotation angle, also the translational motion in x- and y-direction, as well as the out-of-plane rotation, will be considered and included in the model. In this way, the design of the DC-DEA can be properly optimized for applications which differ from soft tentacle arms, and thus require more complex actuation patterns. Motion and forces in all three spatial directions will be evaluated with suitable measurement equipment, and then optimized regarding the geometry as well as the properties of the DE and the applied spacer. Finally, with the aid an adapted simulation model, control of the full 3D motion in space will be achieved.

## Data Availability Statement

The datasets generated for this study are available on request to the corresponding author.

## Author Contributions

SN and RB: experimental results and manufacturing. SC: modeling and simulation results. GR: modeling and paper revision. DN and SS: paper revision.

### Conflict of Interest

The authors declare that the research was conducted in the absence of any commercial or financial relationships that could be construed as a potential conflict of interest.
